# Designing a nurse‐led assessment and care planning intervention to support frail older people in primary care: An e‐Delphi study

**DOI:** 10.1111/jan.15066

**Published:** 2021-10-08

**Authors:** Helen Lyndon, Jos M. Latour, Jonathan Marsden, Bridie Kent

**Affiliations:** ^1^ University of Plymouth Plymouth UK; ^2^ Curtin University Perth Australia

**Keywords:** carers, community care, comprehensive geriatric assessment, frailty, nurse‐led, nurses, nursing, older people, primary care

## Abstract

**Aim:**

To identify and establish expert consensus on important and feasible components of a nurse‐led, comprehensive geriatric assessment (CGA)‐based intervention for community‐dwelling older people who live with frailty.

**Design:**

A three‐round modified e‐Delphi survey.

**Methods:**

An expert panel of 33 UK specialist older people's, primary and community care nurses participated in the three‐round e‐Delphi survey over a 12‐month period in 2017–2018. Data from round 1 were analysed using content analysis. Descriptive statistics were used in the subsequent two rounds to demonstrate convergence of panel opinion and consensus.

**Results:**

In round 1, experts proposed 30 CGA components that were combined with six additional components from a literature review and clustered into six domains. In round 2, components were rated for importance and feasibility. Rating scores for importance were high across all domains, with lower scores for feasibility. Round 3 revealed that 36 components achieved consensus on importance and 11 out of 36 components reached consensus on feasibility.

**Conclusion:**

Based on expert panel opinion, the content of a nurse‐led CGA‐based intervention was established, with the aim of future feasibility testing in a randomized controlled trial.

**Impact:**

This study provides feasible components of a CGA‐based intervention that can be implemented in clinical practice by nurses in partnership with older people who live with frailty. Following further testing and evaluation, the components have the potential to improve clinical outcomes, maximize independence and improve the quality of life for community‐dwelling frail older people.

## 1 INTRODUCTION

The population is ageing and, although this is undoubtedly a success for improved public health and welfare leading to longer life expectancy, it brings with it the challenge of meeting the health and social care needs of higher numbers of older people. By 2050, 1 in 6 people in the world will be over age 65 (16%), up from 1 in 11 in 2019 (9%). In 2018, for the first time in history, persons aged 65 or above outnumbered children under 5 years of age globally. The number of persons aged 80 years or over is projected to triple, from 143 million in 2019 to 426 million in 2050 (United Nations, 2019). In the United Kingdom, remaining life expectancy at age 65 is currently 19.5 years; however, many people experience 10 years of diminished quality of life due predominantly to limiting disability and illness (Mortimer & Green, 2015). Much of this disability and loss of function can be attributed to the development of frailty.

Frailty is a clinical syndrome associated with ageing, which develops through cumulative cellular damage over the life course and leads to progressive disability and loss of independence (Clegg et al., 2013). Biomedical assessment of frailty focusses on the diagnosis and treatment of the clinical syndrome (Hoogendijk et al., 2019), however, this approach fails to capture individuals’ differences and can cause clinicians to neglect peoples’ abilities to participate in their own care and support (Rahman, 2018). The World Health Organisation advocates for an asset‐based model of assessment and support including a holistic, multidimensional approach to managing frailty as a means of preserving function, personhood and independence (World Health Organization, 2015).

Assessment of frailty is mostly undertaken in acute hospitals using a Comprehensive Geriatric Assessment (CGA), led by a geriatrician (Clegg et al., 2013). This assessment and care planning process is acknowledged as the gold standard for the management and prevention of deterioration in frailty (Gladman, 2016). It is a multidimensional, interdisciplinary diagnostic process to determine the medical, psychological and functional capabilities of a frail older person to develop an individualized care plan for treatment and long‐term follow‐up in partnership with the patient and their families (Ellis et al., 2011). However, many older people who live with frailty do not access hospital services, and there is no evidence to indicate that the acute hospital CGA is immediately transferable to community or primary care delivery.

It is not clear whether clinicians in this community setting (including nurses) possess the specialist skills and knowledge to deliver CGA. In addition, concerns have been raised across Europe about the time taken to identify the frail population, conducting a CGA and the additional cost in time and resources to primary care (Shaw et al., 2018). Implementation of primary care frailty management can be problematic because primary care doctors may view frailty screening as a burden in an already challenging workload (Reeves et al., 2018). A holistic, flexible intervention is required that can be delivered by primary care professionals other than GPs and adapted for the individual and their needs. In addition, evaluation of the role of nurses in leading this care model is required.

### 1.1 Background

Beswick et al. (2008) found that the delivery of complex interventions (based on CGA) for older people at home reduces care home and hospital admissions and falls. A systematic review investigating the implementation of one primary care CGA‐based approach noted a lack of an agreed implementation model and concerns of workforce capacity in primary care (Craig et al., 2015). Another review attempted to identify approaches to CGA in primary care and although there were several in existence, the authors highlighted the need for more research into what is feasible for large numbers of the population (Morley et al., 2017).

Professional organizations, such as the British Geriatrics Society (BGS), advocate for a multi‐professional approach to supporting frail patients and point to evidence that nurses and allied health professionals can successfully lead and provide input into the assessment and care planning process (Schadewaldt et al., 2013). Although some authors have evaluated healthcare professionals’ attitudes to frailty assessment and management and found more positive engagement among nurses than other clinicians (Moffatt et al., 2018), the nursing contribution to frailty management is poorly developed, with nurse‐led approaches showing mixed outcomes (Bleijenberg et al., 2017; Schein et al., 2005; Stijnen et al., 2014; Stijnen et al., 2014; Taube et al., 2018). Reviews of some of these studies report a lack of specialist older persons’ knowledge and advanced assessment skills which affected delivery and fidelity to the intervention (Hertogh & Bastiaans, 2016; Hoogendijk et al., 2016). Some authors have suggested that primary care teams require the support of specialist services, such as geriatricians (Hertogh & Bastiaans, 2016), whereas others have employed nurses with advanced assessment and case management skills and reported more positive effects on outcomes (Kono et al., 2016; Rockwood et al., 2000). In addition to advanced clinical skills, several studies have highlighted the importance of a goal‐orientated intervention focussing on person‐centeredness and self‐management. This approach should be built on a caring, supportive relationship between the nurse and patient (Imhof et al., 2012). Therefore, whilst a CGA‐based intervention may be appropriate for primary care delivery, the practicalities of its implementation require further exploration, including which components can and should be led by nurses rather than doctors. This formed the focus of this Delphi study, which is part of a larger programme of research related to the development and implementation of a nurse‐led, CGA‐based intervention.

## 2 THE STUDY

### 2.1 Aim

This study aimed to identify and obtain expert consensus on important and feasible components of a nurse‐led, CGA‐based intervention for community dwelling older people who live with frailty.

### 2.2 Design

The e‐Delphi survey was conducted as the first phase of a mixed‐methods feasibility study to develop and test a nurse‐led assessment and care planning intervention for frail older people in primary care. A modified e‐Delphi technique was used (Foth et al., 2016) with a literature review, expert opinion and achievement of pre‐specified levels of consensus (Keeney, Hasson, & McKenna, 2011). An a priori definition of consensus was agreed by the research team (Jünger et al., 2017) and defined as 75% expert panel agreement that a component met the criteria of ‘fairly important’ or ‘very important’ and ‘fairly feasible’ or ‘very feasible’ was required at round 3 of the survey.

**FIGURE 1:**
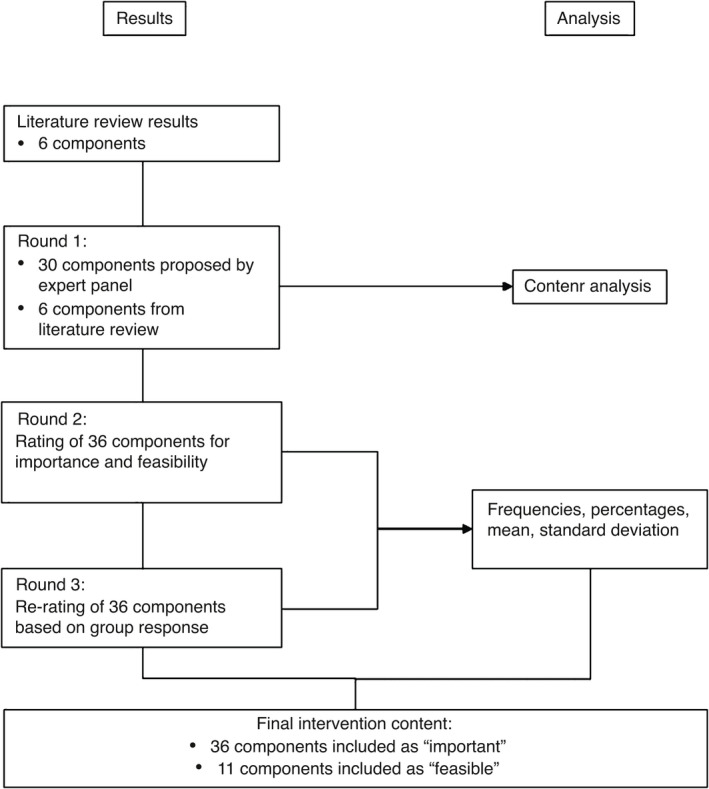
Delphi methods and results

Methods and results are reported in line with the ‘Guidance on Conducting and Reporting Delphi Studies’ (CREDES) (Jünger et al., 2017), which promotes consistency and quality in conducting Delphi studies. Figure 1 summarizes the Delphi process. to provide rigour and transparency in methods, study procedures were planned in detail and piloted whenever possible.

### 2.3 Participants

The research team aimed to recruit at least 20 expert panel members, to form a panel with broad expertise in the care of older people in primary and community care settings. Potential panel members were contacted through the British Geriatrics Society Nurses Council, the Royal College of Nursing Older People's Forum Steering Committee, and the National Health Service (NHS) National Community/Primary Care Nurses Forum. Participating organizations were asked to provide letters of approval and confirm that they would share the survey with their members. An invitation to participate and a participant information sheet was provided. Individuals who chose to participate were asked to confirm consent through the completion of an online consent form as part of the first‐round survey.

### 2.4 Data collection

Three rounds of Delphi procedures were carried out using email invitation and completion of online surveys. Surveys were administered via an online survey platform, ‘SurveyMonkey’ and potential participants were directed to SurveyMonkey (http://www.surveymonkey.com/) with a URL specific to this survey. Email reminders were sent after 1 week and 2 weeks, and withdrawal from the study was offered at all stages.

### 2.5 Survey design and administration

Three members of the research teams designed the round 1 survey. To assess ease of use and understanding of content, the survey was completed initially by two specialist community nurses. They suggested the inclusion of a few examples of potential components. The round 1 survey began with information on the purpose of the study and initial questions related to the demographic characteristics of participants including years qualified as a nurse, specialist area of practice and any specialist qualifications. There was one open‐ended question; ‘Please give your ideas about the components of a CGA that you think are important and will improve clinical outcomes for frail older people in a primary/community setting. Please list as many as you can for example: multidisciplinary team involvement, agreeing a plan of care and support, medication review, environmental assessment, etc.’

Prior to the round 1 survey, a review of the literature, which was conducted to identify what was already known about the content of nurse‐led CGA‐based approaches in primary care. A search of PubMed and CINAHL English language journals from 1990 to 2017 was undertaken to identify articles on the topic. The search strategy combined headings and keywords for ‘comprehensive geriatric assessment’, ‘CGA’, ‘primary care’, ‘community care’, ‘nursing assessment’, ‘care plan’ ‘frail’ and ‘older people’. Information on components of an intervention were extracted and listed, and any not suggested by the expert panel were added to the round 2 survey.

The round 2 survey consisted of 30 components of CGA suggested as important by the panel members and six additional components derived from the literature review. Panel members were asked to rate each component on two issues: importance and feasibility. Importance was rated on a 5‐point, Likert‐type scale ranging from 1 is ‘not important at all’ to 5 is ‘very important’. The feasibility scale was a 5‐point, Likert‐ type scale ranging from 1 is ‘not feasible’ to 5 is ‘very feasible’. Importance in this case related to how important was it that this component formed part of the CGA‐based approach and feasibility related to the practicality of implementing this component in community and primary care practice. The last question in this survey round was an open‐ended question asking if there were any missing components that could be included in the next round.

The round 3 survey listed the 36 components from round 2 with the aggregated results (frequency and percentage) for each component from round 2. Data were presented back to the panel along with the same rating scales so that panel members had the opportunity to re‐rate based on the group response in round 2.

### 2.6 Ethics considerations, confidentiality and data security

Ethics approval was granted by the University of Plymouth Faculty Research Ethics and Integrity Committee (Reference Number: 18/19‐1027). The online platform used to administer the surveys has data security and privacy policies in place (SurveyMonkey, 2020). Information systems and technical infrastructure are hosted in accredited data centres. Access to technology resources is only permitted through secure connectivity and requires multi‐factor authentication. All data are encrypted, and all responses to surveys are private by default.

Expert panel members were assured of anonymity in the participant information sheet. Internet protocol addresses were used to contact panel members who could not be identified in the process, and individual responses were unknown to other panel members. Panel members were asked to sign an informed consent form including agreement to the use of email addresses to contact with subsequent survey rounds.

### 2.7 Data analysis

Statistical analysis and definition of consensus were planned and agreed prior to data collection. Panel members’ demographic characteristics were reported by frequencies and percentages. Data from the open‐ended question in round 1 were analysed using content analysis (Hasson et al., 2000). In round 2, frequencies and percentages for all ranking scales were calculated prior to being presented back to the panel in round 3. In addition to frequencies being derived, means and standard deviations for ranking scores were calculated to assess convergence of opinions from round 2 to round 3. Final consensus figures (percentage consensus for each component) were calculated for reporting after round 3.

There is no specific guidance available for acceptable response rates in Delphi studies. Some Delphi studies relating specifically to older people's care have not reported response rates (Goldberg et al., 2016; Mahoney et al., 2017). However, others have reported between 75% (Rodríguez‐Mañas et al., 2013) and 92% (Jeffs et al., 2017). Because of the iterative process of Delphi studies, there is the potential for panel members to withdraw after subsequent rounds, which can lead to response bias if attrition is significant (Evans, 1997). Some authors recommend that a 70% response rate is necessary for each round to maintain rigour (Sumison, 1998). In this study, a response rate of 70% was anticipated to the rounds 2 and 3. To encourage consensus, three reminders to complete the survey were sent to the panel members.

### 2.8 Validity, reliability and rigour

No verified survey instrument was available to use in this study. To validate the components of the intervention suggested by panel members in the round 1 exploratory survey, the team conducted a review of the literature to identify current evidence about the content of nurse‐led CGA‐based approaches in primary/community care. Information on components of an intervention were extracted and listed. Panel responses in round 1 were compared with the literature, and any additional components not suggested by the panel were added to the round 2 survey.

## 3 RESULTS

### 3.1 Expert panel demographics

A total of 75 volunteers, all experienced nurses who, at the time of the e‐Delphi, worked with older people in primary and community healthcare settings were invited to participate. Of these, 33 responded to the first round, and one respondent withdrew from subsequent rounds, resulting in 32 participants for rounds 2 and 3. Years since qualification ranged between 6 and 41 years with a mean of 28 years (Table 1). The majority of responses were received from participants who worked in either older people's nursing (*n* = 14) or community nursing (*n* = 13) with a minority working in general practice nursing (n = 1). Other specialities included academia, intermediate care and mental health nursing. Of the expert panel members, 36.5% held a specialist community nursing qualification, 27% held a specialist older people's nursing qualification, whereas 33% had no specialist qualification. None of the panel held a specialist general practice nursing qualification. Response rate to the round 2 survey was 72% (23 out of 32), and the round 3 survey achieved a 91% response rate (21 out of 23).

**TABLE 1:** Demographics of the expert panel

Demographic characteristics	*n* (%)
Nursing speciality	
Older peoples’ nursing	14 (42.5)
General practice nursing	1 (3.0)
Community nursing	13 (39.5)
Other (please specify)	5 (15.0)
Years qualified as a nurse	
0 to10	3 (10.0)
11 to 20	4 (12.0)
21 to 30	9 (27.0)
31 to 40	16 (48.0)
More than 40	1 (3.0)
Specialist nursing qualification	
Older peoples’ nursing qualification	9 (27.0)
Community nursing qualification	12 (36.5)
Practice nursing qualification	0 (0.0)
No specialist qualification	11 (33.0)
No answer given	1 (3.0)

### 3.2 e‐Delphi round 1

Content analysis generated an initial 35 components suggested by the expert panel. These were aggregated into 30 components and grouped into six domains, which were as follows:

1. Frameworks/care structures; the organizational procedures, which are required to support the delivery of the intervention
2. Home/family/safety assessment; including functional, environmental, social and carers needs assessments
3. Personalized care and support planning; supporting self‐management and person‐centred care with the development and ongoing evaluation of a plan of care
4. Long‐term condition management; identification of problems/deficits related to long‐term conditions/multimorbidity including medication review
5. Physical health assessment; including frailty, nutrition/hydration and sexual health
6. Mental health assessment; including cognition and mood

Six additional components were incorporated from the literature review. These components were a system for information gathering; a shared care record; listening to the patient's story as part of personalized care and support planning; assessment of pain; assessment of vision, hearing and dentition and assessment of bladder and bowel function. The combination of these components and those from the experts resulted in a round 2 survey of 36 components clustered in the six domains (Table 2).

**TABLE 2:** Components identified in round 1

	Components	Number of responses
	**Frameworks/care structures**	
1	Multi‐disciplinary team discussion/review	8
2	Coordinated multidimensional assessment and care with an identified lead clinician/case manager	5
3	A competent, well‐trained workforce who can deliver an assessment and care planning intervention	3
4	A timely response to crises	1
5	A system for data/information gathering, e.g. past medical history, social circumstances, family history	*
6	A shared care record	*
	**Home/family/safety assessments**	
7	Environmental assessment including housing and equipment aimed at maximizing independence	11
8	Assessment of social support including financial concerns, benefits entitlement, social isolation	8
9	Assessment of functional ability and activities of daily living including re‐ablement potential	5
10	Assessment of falls risk	3
11	Assessment of carer's needs	3
12	Determining spiritual needs and support systems	1
13	Exploring opportunities for employment/education/hobbies	1
	**Personalized care and support planning**	
14	Agreeing and formulating a plan together based on shared decision‐making and the preferences of the individual: working the partnership	10
15	Safeguarding this contract by documenting it in a co‐created care or support plan: personalized care and support planning	10
16	Monitoring response to the care and support plan	10
17	Review and revising of the care and support plan	10
18	Empowerment and self‐management and enabling behavioural change	6
19	Determining advance care preferences	4
20	Establishing the patient's personal goals and where support is needed (person centred care)	4
21	Assessment of resilience and coping mechanisms – an asset‐based approach	3
22	Escalation/contingency planning: actions for when the patient's condition deteriorates	2
23	Assessment of patient's ability to actively participate in care and planning	2
24	Establishing an individual's narrative by active listening/appreciative enquiry	*
	**Long‐term condition management**	
25	Medication review including ability to self‐administer, concordance and de‐prescribing	10
25	Advanced clinical assessment skills – physical examination and ordering investigations	6
27	Problem/deficit identification	3
28	Optimizing management of long‐term conditions/multimorbidity	1
	**Physical health assessments**	
29	Assessment for the presence and severity of frailty	2
30	Assessment of nutritional status including hydration	1
31	Sexual health assessment	1
32	Assessment of pain	*
33	Assessment of vision, hearing and dentition	*
34	Assessment of bladder and bowel function	*
	**Mental health assessments**	
35	Assessment of cognition	6
36	Assessment of mood and psychological well‐being	6

* Component taken from literature review.

### 3.3 e‐Delphi round 2

The mean of all expert panel scores was calculated for each component and then combined to give a mean score for each domain. When analysed in the six domains, mean scores for importance were high across all components and lower for feasibility except for the frameworks/care structures domain, which were high for both importance (mean 4.8; SD 0.06) and feasibility (mean 4.8; SD 0.06). All other domains had mean scores ranging from 4.4 (SD 0.50) to 4.59 (SD 0.11) for importance and between 3.47 (SD 0.25) and 4.59 (SD 0.33) for feasibility. No additional components were suggested by panel members. Full results with frequencies, percentages, mean scores and standard deviations for all components in round 2 are presented in Supplementary Information 1.

### 3.4 e‐Delphi round 3

**FIGURE 2:**
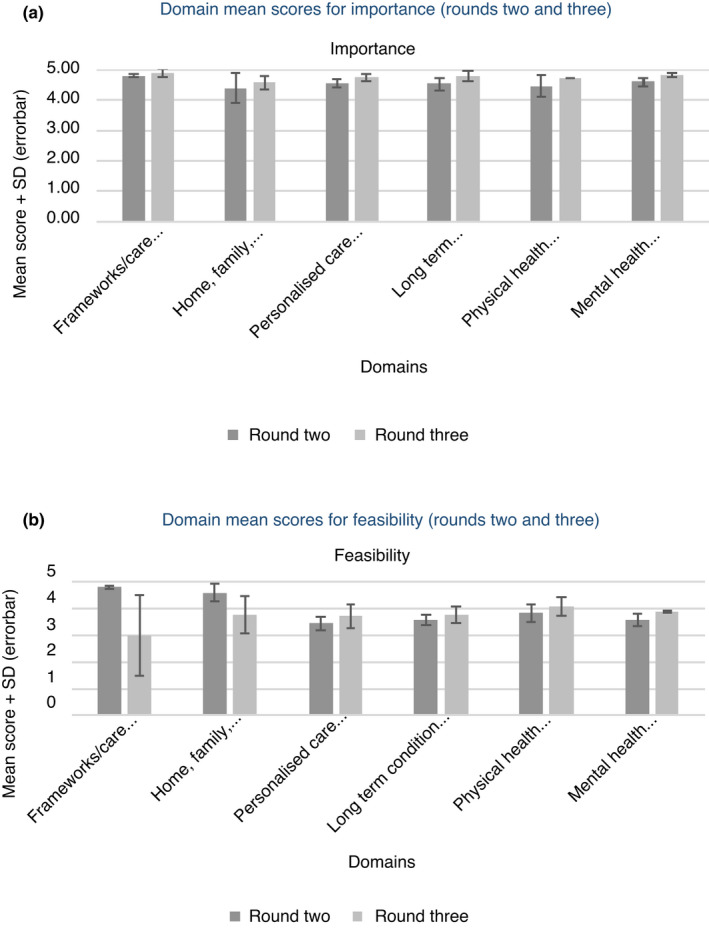
(a) Domain mean scores for importance (rounds two and three). (b) Domain mean scores for feasibility (rounds two and three)

Domain mean scores for both rounds are presented in Figure 2a,b revealing increasing mean scores and convergence of opinion across the rounds. Generally, mean scores for importance remained high across all domains, with lower scores for feasibility. Scores relating to the domain of frameworks/care structures were higher in round 3 (mean 4.88; SD 0.13) than in round 2 (mean 4.80; SD 0.06) for importance, but with a larger standard deviation in round 3, indicating more variability in scoring. However, feasibility scores reduced and had larger standard deviations from round 2 (mean 4.8; SD 0.06) to round 3 (mean 3.0; SD 1.5) indicating less consensus on feasibility of these components. The component relating to the need for a shared care record in this domain strongly influenced the overall mean score (mean 2.83; SD 0.75). Mean scores relating to the other four domains all increased from round 2 to round 3 with variable standard deviations; personalized care and support planning (importance: 4.55; SD 0.12 to 4.74; SD 0.12, feasibility: 3.47; SD 0.25 to 3.7; SD 0.44), long term condition management (importance: 4.53; SD 0.19 to 4.79; SD 0.18, feasibility: 3.59; SD 0.19 to 3.78; SD 0.31), physical health assessments (importance: 4.47; SD 0.36 to 4.72; SD 0.00, feasibility: 3.85; SD 0.32 to 4.10; SD 0.35) and mental health assessments (importance: 4.59; SD 0.11 to 4.82; SD 0.07, feasibility: 3.57; SD 0.23 to 3.9; SD 0.03). This may indicate overall convergence of opinion across rounds but with small numbers of outlying opinions, which affected standard deviation. Results with frequencies, percentages, mean scores and standard deviations for all components are presented in Supplementary Information 2.

### 3.5 Panel consensus

Following round 3, all 36 components met consensus on importance, but only 11 out of the 36 components reached consensus on feasibility at the pre‐defined level of 75% panel agreement (Table 3). In the frameworks/care structure domain, all components met the consensus threshold for importance (range 90.5%−100%), but four out of the six did not reach consensus on feasibility. These were a competent, well‐trained workforce (47.6%), a system for data/information gathering (47.6%), a shared care record (57.8%) and a timely response to crisis (19.0%).

**TABLE 3:** Final percentages for each component (importance and feasibility)

	Components	Importance	Feasibility
	**Frameworks/care structures**		
1	Multi‐disciplinary team discussion/review	100%	81.0%
2	Coordinated multi‐dimensional assessment and care with an identified lead clinician	100%	76.2%
3	A competent, well‐trained workforce who can deliver the intervention	95.3%	47.6%
4	A timely response to crises	90.5%	19.1%
5	A system for data/information gathering, e.g. past medical history, social circumstances	100%	47.6%
6	A shared care record	95.2%	57.8%
	**Home/family/safety assessments**		
7	Environmental assessment aimed at maximizing independence	95.2%	52.4%
8	Assessment of social support including financial concerns, social isolation	95.2%	47.6%
9	Assessment of functional ability and activities of daily living including reablement potential	95.2%	85.7%
10	Assessment of falls risk	100%	81.0%
11	Assessment of carer's needs	100%	66.7%
12	Determining spiritual needs and support systems	95.2%	57.1%
13	Exploring opportunities for employment/education/hobbies	81.0%	38.1%
	**Personalized care and support planning**		
14	Agreeing and formulating a plan together based on shared decision‐making	90.5%	57.1%
15	Safeguarding this contract by documenting it in a co‐created care or support plan	85.7%	33.3%
16	Monitoring response to the care and support plan	85.7%	42.9%
17	Review and revising of the care and support plan	95.2%	61.9%
18	Empowerment and self‐management and enabling behavioural change	95.2%	33.3%
19	Determining advance care preferences	100%	71.4%
20	Establishing the patient's personal goals and support needed (person‐centred care)	95.2%	81.0%
21	Assessment of resilience and coping mechanisms – an asset‐based approach	95.2%	33.3%
22	Escalation/contingency planning: actions for when the patient's condition deteriorates	100%	61.9%
23	Assessment of patient's ability to actively participate in care and planning	85.7%	76.2%
24	Establishing an individual's narrative by active listening/appreciative enquiry	90.5%	52.4%
	**Long‐term condition management**		
25	Medication review including ability to self‐administer, concordance and de‐prescribing	100%	81.0%
26	Advanced clinical assessment skills – physical examination and ordering investigations	90.5%	57.2%
27	Problem/deficit identification	95.2%	71.4%
28	Optimizing management of long‐term conditions/multimorbidity	100%	71.4%
	**Physical health assessments**		
29	Assessment for the presence and severity of frailty	90.5%	81.0%
30	Assessment of nutritional status including hydration	100%	85.7%
31	Sexual health assessment	81.0%	28.6%
32	Assessment of pain	100%	95.2%
33	Assessment of vision, hearing and dentition	100%	66.7%
34	Assessment of bladder and bowel function	100%	81.0%
	**Mental health assessments**		
35	Assessment of cognition	100%	71.4%
36	Assessment of mood and psychological well‐being	100%	66.7%

In the home/family/safety assessment domain, all components met the consensus threshold for importance (range 81.0%−100%), but five out of the seven did not reach consensus on feasibility. These were environmental assessment (52.4%), assessment of social support (47.6%), assessment of carer need (66.7%), determining spiritual needs (57.1%) and exploring opportunities for employment/education/hobbies (38.1%).

In the domain of personalized care and support planning, all components met the consensus threshold for importance (range 85.7%−100%), but nine out of the eleven did not reach consensus on feasibility. These were formulating a personalized care and support plan (PCSP) (57.1%), documenting in a co‐created PCSP (33.3%), monitoring response (42.9%), review of the PCSP (61.9%), empowerment and self‐management (33.3%), determining advanced care preferences (71.4%), assessment of resilience and coping mechanisms (33.3%), escalation/contingency planning (61.9%) and establishing the narrative (52.4%).

All four components in the long‐term condition management domain met the threshold for consensus on importance (range 90.5%−100%) while three of these did not achieve consensus on feasibility; advanced clinical assessment skills (51.2%) and problem/deficit identification and optimizing long‐term condition management (both 71.4%).

In the domain of physical health assessments, all components met the consensus threshold for importance (range 80.1%−100%), and two out of the six did not reach consensus on feasibility; assessment of vision, hearing and dentition (66.7%) and sexual health assessment (28.6%). Finally, in the domain of mental health assessments, both components of assessment of cognition and assessment of mood met the consensus threshold for importance (100%), but not for feasibility scoring 71.4% and 66.7%, respectively.

## 4 DISCUSSION

This e‐Delphi study identified and reached consensus on important and feasible components of a nurse‐led CGA‐based intervention for community dwelling older people who live with frailty. Following three rounds of surveys, the expert panel identified what they considered to be both important and feasible components of the care model, and consensus at the required level for importance was reached for all suggested components. The important components were similar to those contained in a primary care CGA toolkit published in 2019 (Turner et al., 2019). However, the panel did not think the majority of the components were feasible to deliver in current primary and community care. It appeared that the expert panel had concerns about the time and infrastructure available to complete a CGA or that there may be a lack of specialist skills.

There was clear concern demonstrated in the low feasibility scores for the existence of a shared care record to enable information gathering and sharing across organizations and the components that relate to personalized care and support planning. In 2013, the National Collaboration for Integrated Care and Support published its report ‘Integrated Care and Support: Our Shared Commitment’ (National Collaboration for Integrated Care & Support, 2013) which stated the government's pledge to end institutional divisions and provide seamless health and social care for older people. The panel members’ responses highlighted the importance of a competent, well‐trained workforce and demonstrated concerns about the feasibility of working across organizational boundaries in partnership to develop personalized plans of care. Studies have demonstrated the value of multi‐professional involvement and a shared care record (Garrard et al., 2020; Phelan et al., 2007). The reality in primary care is still far from the strategic vision of integrated care for older people.

Other components that were thought not feasible relate to the possession of specific skills by primary care nurses. The panel thought nurses would not be able to assess carer's needs, conduct environmental assessments and determine preferred place of care. They also doubted the feasibility of nurses with advanced assessment skills. This is borne out by the literature where some studies have reported a lack of specialist older people knowledge and skills amongst primary care nurses (Hertogh & Bastiaans, 2016; Hoogendijk, 2016).

Notwithstanding the concerns already discussed, it was encouraging to see that specific components from other domains were thought to be important and feasible in a nurse‐led approach. These were establishing the diagnosis and severity of frailty and assessment of functional ability including re‐ablement, falls risk, pain, medication adherence and optimization, nutritional status (including hydration) and bladder and bowel function. This is important information in informing the design of a nurse‐led, CGA‐based intervention as, to date although studies of primary/community care–based CGA have demonstrated some benefits relating to clinical outcomes and acceptance by patients (Fenton et al., 2006; Hermush et al., 2009; Phelan et al., 2007) they have not, however, specifically detailed the content of the intervention under study. Transparency about specific content would enable further evaluation or assessment in clinical practice.

It is interesting to note that assessment components demonstrated a holistic approach and were not limited to biomedical interventions. This is in line with the concept of intrinsic capacity, which has been adopted by the World Health Organisation as a quantifiable measure of healthy ageing (World Health Organization, 2015) and a composite measure of all physical and mental capabilities of an older person (Woo, 2019). This construct moves away from the biomedical approach of diagnosing and treating diseases, and towards assessment of body functions as a ‘holistic entity’ (Cesari et al., 2018, p.3), supporting prevention or managing deterioration, aiming always to preserve function and independence. This is more in tune with older people's perceptions of becoming frail in terms of everyday tasks and how it feels if these tasks start to become difficult to complete, thus eroding independence and wellbeing (Britain Thinks, 2015).

The panel members had reservations about whether completion of CGA in current primary healthcare practices is possible, and this reflects global concerns about shortage of primary healthcare professionals (World Health Organization, 2013) and the debate about how capacity and clinical quality can be increased by new models of care provided closer to home (Elkan et al., 2001). They also echo results of other studies that highlight the perceived challenges to the delivery of primary care‐based CGA (Craig et al., 2015; Monteserin et al., 2010; Stijnen et al., 2014). This may be an opportunity to examine which clinicians are best placed to provide care and support to frail older people in a way that can increase capacity in the primary/community care team and provide a more convenient approach for patients who would struggle to attend secondary care. A new model may include the substitution of nurses where care and treatment has previously been provided by doctors, for example, as an alternative to geriatrician‐led CGA.

Ensuring the most appropriate clinician delivers care and support is an ongoing debate, with increasing acceptance that care for older people with complex needs can be led by nurses. Three systematic reviews have reported that care provided by nurses is of equal quality to care provided by primary care doctors (Horrocks et al., 2002; Laurant et al., 2005; Martínez‐González et al., 2014). Recently, the BGS affirmed that nurses can lead CGA‐based interventions in primary care as they are ‘are well placed to manage the complexity of assessment in an efficient way drawing together the different strands to coordinate a personalized treatment plan’ (Turner et al., 2019, p. 4). Turner et al. also emphasize that nurses have a duty to act as patient advocate set out in their codes of conduct and are expert in enabling shared decision‐making. Given that CGA is a multi‐dimensional assessment and care planning process, it has been advocated that multiple clinicians should be involved and that nurses should coordinate and lead the process ensuring best use of scarce resources and targeting of this approach at those who will most benefit (Schadewaldt et al., 2013).

### 4.1 Study limitations

Older people and their carers were not included in the expert panel, and this was a limitation of the study as the expert panel did not necessarily reflect their views on what was important to them. To address this deficit, the Delphi findings were later shared with a research stakeholder group made up of older people, carers and clinicians to consult with them the results of this study and on the final content and delivery methods. Unfortunately, there are no qualitative data that can explain the reasons why participants felt that several components were not considered to be feasible and so were not included in the emergent nurse‐led CGA model.

## 5 CONCLUSION

This e‐Delphi study developed consensus on important and feasible components of a nurse‐led, CGA‐based intervention in primary care. The study indicates which components of traditional CGA can be effectively delivered in primary care, by non‐medical practitioners, as well as those elements which may not be feasible in practice. The intervention now requires further evaluation in real life clinical practice and will be tested in a feasibility randomized controlled trial in the next phase of this research.

## 6 Funding statement

Helen Lyndon has been awarded a National Institute of Health Research Clinical Doctoral Research Fellowship which funded this study.

## CONFLICT OF INTEREST

No conflict of interest has been declared by the authors.

## AUTHOR CONTRIBUTIONS

All authors have agreed on the final version and meet at least one of the following criteria (recommended by the ICMJE*):

1. substantial contributions to conception and design, acquisition of data or analysis and interpretation of data
2. drafting the article or revising it critically for important intellectual content.

### PEER REVIEW

The peer review history for this article is available at https://publons.com/publon/10.1111/jan.15066.

## Supporting information

Supplementary Material

Supplementary Material
